# How sensitive are avoidable emergency department attendances to primary care quality? Retrospective observational study

**DOI:** 10.1136/bmjqs-2020-011651

**Published:** 2020-11-03

**Authors:** Beth Parkinson, Rachel Meacock, Kath Checkland, Matt Sutton

**Affiliations:** 1 Health, Organisation, Policy and Economics (HOPE) Group, Centre for Primary Care & Health Services Research, University of Manchester, Manchester, Greater Manchester, UK; 2 Melbourne Institute of Applied Economic and Social Research, Faculty of Business and Economics, The University of Melbourne, Parkville, Victoria, Australia

**Keywords:** emergency department, general practice, health policy

## Abstract

**Background:**

Improvements in primary care quality are often proposed as a solution to rises in emergency department (ED) attendances. However, there is little agreement on what constitutes an avoidable attendance, and the relationship between primary care quality and ED demand remains poorly understood.

**Objective:**

To estimate the size of the associations between primary care quality and volumes of ED attendances classified as avoidable.

**Methods:**

Retrospective observational study of all attendances at EDs in England during 2015/2016, applying three definitions of avoidable attendance. We linked practice-level counts of attendances to seven measures of primary care access, patient experience and clinical quality for 7521 practices. We used count data regressions to associate attendance counts with levels of quality. We then calculated proportions of attendances associated with levels of primary care quality below the national average.

**Results:**

Attendance volumes were negatively related to three of the seven quality measures. Incidence rate ratios (IRRs) for all attendances associated with 10 percentage-point differences in quality were 0.987 for clinical quality and 0.987 for easy telephone access and 0.978 for ability to get an appointment. These associations were relatively stronger for narrower definitions of avoidable attendances (for the narrowest definition, IRRs=0.966, 0.976 and 0.934, respectively) but represented fewer attendances in absolute terms. 341 000 (2.4%) attendances were associated with levels of primary care quality below the national average in 2015/2016.

**Conclusion:**

ED attendances are sensitive to primary care quality, but magnitudes of these associations are small. Attendances are much less responsive to differences in primary care quality than indicated by estimates of the prevalence of avoidable attendances. This may explain the failure of initiatives to reduce attendances through primary care improvements.

## Introduction

Increases in the volume of emergency department (ED) attendances have put pressure on healthcare services worldwide, resulting in adverse impacts on the quality and safety of patient care.^1^ It is often suggested that many attendances are avoidable and that these patients could have been more appropriately treated elsewhere, most often in a primary care setting.^2^ Several interventions have been introduced to manage demand for EDs, with improving accessibility of primary care services during and outside of normal working hours a key strategy.^3^ Such policies assume that a proportion of current attendances could have been avoided if access to primary care was improved. However, the relationship between the quality of primary care services and demand for ED services is poorly understood.

A recent systematic review of studies assessing the effect of primary care practice-level factors on ED attendances found that continuity of care and access to general practice were generally associated with fewer ED attendances, while evidence relating to quality of care was limited and inconclusive.^4^ However, the included studies often focused on specific geographical areas or patient populations. To replicate the findings from their systematic review, Tammes and colleagues conducted both cross-sectional^5^ and longitudinal studies^6^ using data from across England. Both studies showed some evidence of an association between accessibility of primary care and the volume of attendances that were self-referred and did not result in an admission. However, another recent study in England found no association between patient experience of general practice and the volume of ED attendances.^7^

There have been many attempts to calculate the proportion of ED attendances that are ‘avoidable’, yet there is currently no agreement regarding what constitutes an avoidable attendance, and no standardised definition exists.^2 8 9^ Definitions generally attempt to capture attendances that are non-urgent, are self-presentations, receive care that could be delivered by a general practitioner (GP) and do not require admission.^2^ However, different criteria have been used to identify these patients empirically.

The lack of agreement as to what constitutes an avoidable ED attendance likely explains the inconsistency in the attempts to link primary care quality with the volume of avoidable attendances. It also means there is no reliable estimate of the magnitude of the problem, that is, the number of ED attendances that are sensitive to primary care quality.

In this paper, we aimed to estimate the size of the associations between primary care quality and volumes of ED attendances classified as avoidable. Furthermore, we quantify the proportion of ED attendances that are associated with levels of primary care quality below the national average. We extend the existing literature by using three definitions of avoidable attendances and several aspects of primary care quality. Thus, we examine comprehensively the sensitivity of the volume of ED attendances to primary care quality.

## Methods

### Study setting: emergency care in England

In England, patients register with a single general practice of their choice to access appointments for both routine and urgent primary care. GPs act as gatekeepers to specialist services provided by hospitals. While general practices do provide out-of-hours services, most appointments occur during normal working hours.

If patients perceive they need more urgent or more specialised services than are immediately available from their GP, they can obtain urgent care from an ED or walk-in centre. There are three categories of ED. The majority of emergency attendances are at type 1 departments, which are consultant-led with full resuscitation facilities open 24 hours a day.^10^ Patients cannot book an appointment, but there is a maximum waiting time target of 4 hours, which should be met for 95% of patients.^11^

General practices are paid by capitation. EDs are reimbursed per attendance under an activity-based financing scheme.^12^ The costs of attendances are borne by local third-party payer organisations, Clinical Commissioning Groups. General practices thus have little direct financial incentive to avoid patients attending EDs, although most Clinical Commissioning Groups will intervene if practice rates fall outside of norms.

Primary and secondary care are provided free at point of use, funded through taxation. This makes England a useful setting for examining the relationship between primary care quality and ED attendances, as it is not confounded by variations in insurance coverage.

### Data

#### Outcome variables: ‘avoidable’ ED attendances

We used patient-level data on attendances at EDs in England for the financial year 1 April 2015–31 March 2016. These records are taken from Hospital Episode Statistics (HES), an administrative data set capturing all hospital activity in England.^13^ We focused on attendances at major (type 1) EDs. To control for the availability of other emergency care services in the area, we included the practice-level rate of attendance at type 2 (single specialty centres), type 3 (minor injury units) and type 4 (walk-in centres) EDs.

We identified three definitions which had been used to retrospectively identify potentially avoidable attendances in previous studies in England.^14^ They vary in the number and strictness of the criteria applied:

1. ‘Self-referred discharges’: patients who were ‘self-referred and sent home (discharged) (from the ED) either with no follow-up or follow-up treatment to be provided by their GP’ as in Tammes *et al*.^5 6^
2. ‘GP treatable’: ‘first attendance with some recorded treatments or investigations, all of which may have been reasonably provided by a GP, followed by being sent home or to GP care’. This is currently used in the online data tool on ‘unnecessary Accident and Emergency (A&E) attendances’ published by NHS Digital, the national NHS information organisation in England.^15^
3. ‘No treatment and discharged’: patients who were ‘self-referred; was an initial (rather than follow-up) attendance for this condition; received no investigation and either no treatment or ‘guidance/advice only’; and were sent home with either no follow-up or follow-up with primary care’ as in McHale *et al*.^16^

We generated indicators to identify attendances belonging to these three definitions by mapping the characteristics described in the studies to variables in HES attendance records. Online supplemental appendix 1 provides details of this process.

10.1136/bmjqs-2020-011651.supp1 Supplementary data

We then used the general practice identifier to generate practice-level counts of each type of attendance, as well as total attendances (online supplemental appendix 2 provides details of the aggregation process).

#### General practice quality variables

We included explanatory variables measuring realised access, continuity of care, clinical quality and patient experience, obtained from the General Practitioner Patient Survey (GPPS) and the Quality and Outcomes Framework (QOF). Online supplemental appendix 3 provides a description of all explanatory variables, including sources and how variables were constructed.

The GPPS is a biannual postal survey conducted on behalf of NHS England. Participants are sampled from practice registration lists to collect patients’ views and experiences of the services provided.^17^ We use data from the July 2016 publication that contains data collected from July to September 2015 and from January to March 2016. Across these two waves, 2 148 791 surveys were distributed with a response rate of 38.9% (836 312 returned surveys).^18^

From the GPPS, we obtained proportions of patients who: would recommend the practice to someone who has just moved to their local area; reported good overall experience of their GP surgery; found it easy to get through to their GP surgery on the phone; were able to make an appointment to see or speak to someone when they wanted to; were able to make a same day appointment; and, among patients with a preferred GP, were able to speak to their preferred GP always or a lot of the time.

To ensure representativeness of practices’ registered populations, responses were published weighted by characteristics of the practice list.^18^ The only measure for which we did not use the weighted version is ‘the proportion of patients that were able to speak to their preferred GP always or a lot of the time’, as the weighted version of this variable is missing for practices with low response rates to this question (n=397). We examined the sensitivity of the results to using the weighted versus unweighted version of this measure. We also controlled for the GPPS response rate at each practice.

The QOF is a national scheme that provides financial incentives for general practices to improve their quality of care.^19^ Practices score points for achieving targets on a range of indicators. The QOF contains three domains: clinical, public health, and public health additional services. We used the proportion of clinical QOF points achieved in 2015/2016 as an indicator of clinical quality.

We tested for multicollinearity among practice quality and accessibility variables using the variance inflation factor. For all variables, these inflation factors were under the commonly used threshold for high multicollinearity of 10.^20^

#### Practice and patient population characteristics

We included several characteristics of practices and their registered populations as potential predictors.

We adjusted the attendance counts for the size of registered patient populations and control for the following characteristics: age distribution (proportion 0–4 years, 5–15 years, 45–54 years, 55–64 years, 65–74 years, 75–84 years and 85+ years),^21^ income deprivation,^22^ proportion unemployed^23^ and proportion whose ethnic group was UK white.^23^ We also controlled for differences in the health of the registered populations by including prevalence rates of five health conditions, obtained from the QOF^24^: chronic obstructive pulmonary disease, heart failure, asthma, atrial fibrillation, and coronary heart disease.

We controlled for practice size using the average number of GPs working at the practice over the year.^25^ We also included several geographical characteristics: a rural indicator,^26^ indicators for 13 NHS regions^27^ and distance between each GP practice and the nearest type 1 ED in England as an estimate of patients’ travel time to the nearest ED.

A full description of how all explanatory variables were constructed and sourced is provided in online supplemental appendix 3. Data on the outcome measures and practice factors were merged using GP practice identifier codes (see online supplemental appendix 2 for full details of the merging process).

### Statistical methods

We used negative binomial regressions to model the three different counts of avoidable ED attendances as a function of primary care quality, population and practice characteristics. We also modelled the count of all ED attendances for comparison. The negative binomial model is a generalisation of the Poisson model, including an additional parameter to account for overdispersion, a common feature of healthcare use data.

We included practice list size as the exposure variable; its coefficient is constrained to unity so that the coefficients in the model can be interpreted as the effect on the annual rates of attendance per registered patient. Coefficients are reported as incidence rate ratios (IRRs), meaning that values greater than 1 indicate a positive association with the volume of attendances, while values less than 1 indicate a negative association. For continuous variables, the IRR can be interpreted as a 100(IRR−1)% difference in the rate of attendances per one-unit difference in the explanatory variable, while for categorical variables (eg, deprivation decile), this would be a 100(IRR−1)% difference relative to the reference group. We scaled all the quality variables so that one-unit differences represent differences of 10 percentage points.

We used robust SEs, clustered at the Clinical Commissioning Group level. Since each model considers the annual rate of attendance per registered patient and coefficients are reported as IRRs, we are able to directly compare the magnitudes of the coefficients across different definitions of attendance. The coefficients represent relative measures of the risk of attendance incidents associated with measures of primary care quality. If the three definitions of ‘avoidable’ attendances accurately capture patients that are either divertible or deferrable to primary care, then these measures should be more sensitive than total attendance volumes to primary care quality. This should be both in terms of the magnitude and statistical significance of the coefficients.

We then explored the sensitivity of ED attendance volumes to primary care quality by estimating the number of attendances that were associated with below-average levels of the seven primary care quality variables. This is calculated as the difference between the numbers of attendances predicted by the models using (1) the observed levels of quality recorded for each practice and (2) replacing values of the quality variables below the national average with the national average. These estimates therefore represent the annual number of attendances in England associated with below-average primary care quality.

#### Sensitivity analyses

We ran three sensitivity checks. First, we removed practices within 20 km of the borders with Scotland or Wales (n=323) as we were unable to measure attendances at EDs outside of England. Second, we included ratings from the Care Quality Commission (CQC), the independent regulator of health and social care services in England, as an additional measure of primary care quality. CQC inspections are carried out at least every 5 years, and so ratings do not necessarily correspond to quality during the year of the analysis. Third, we tested the sensitivity of the results to using weighted rather than unweighted ‘proportions of patients able to see their preferred GP’.

## Results

The mean number of ED attendances was 257 per 1000 registered patients (table 1). On average, there were 94 attendances per 1000 registered patients classified as avoidable using definition 1 (self-referred discharges); 34 classified as avoidable using definition 2 (GP treatable), and 16 classified as avoidable using definition 3 (no treatment and no follow-up attendances).

**Table 1:** Descriptive statistics on the 7521 general practices included in the analysis

Rates of ED attendance per 1000 population	Mean	SD	25th percentile	Median	75th percentile
Total ED attendances	256.5	89.8	195.1	247.6	311.4
Definition 1: self-referred discharged (Tammes *et al)* 5 6	93.6	50.4	56.7	86.9	124.0
Definition 2: GP treatable (NHS Digital)^15^	33.7	23.6	17.2	28.2	44.0
Definition 3: no treatment and no-follow up (McHale *et al*)^16^	15.5	12.8	6.8	12.5	20.5
Primary care quality measures					
Proportion of clinical Quality and Outcomes Framework points achieved	0.954	0.070	0.943	0.977	0.994
Proportion of patients who would recommend the practice	0.774	0.124	0.701	0.793	0.869
Proportion of patients reporting good overall experience of GP surgery	0.852	0.092	0.803	0.870	0.921
Proportion of patients who could see their preferred GP	0.620	0.173	0.500	0.630	0.750
Proportion of patients reporting easy phone access	0.731	0.168	0.625	0.761	0.865
Proportion of patients able to get an appointment to see or speak to someone	0.847	0.081	0.801	0.860	0.906
Proportion of patients who were able to get same day appointment	0.367	0.145	0.258	0.350	0.458
Control variables					
Size of the registered population	7459	4444	4086	6629	9965
Proportion of registered population aged 0–4 years	0.060	0.016	0.049	0.058	0.069
Proportion of registered population aged 5–15 years	0.126	0.027	0.112	0.124	0.139
Proportion of registered population aged 16–44 years	0.388	0.086	0.333	0.373	0.426
Proportion of registered population aged 45–54 years	0.143	0.021	0.134	0.146	0.156
Proportion of registered population aged 55–64 years	0.113	0.026	0.097	0.116	0.130
Proportion of registered population aged 65–74 years	0.093	0.035	0.068	0.094	0.117
Proportion of registered population aged 75–85 years	0.055	0.022	0.039	0.055	0.069
Proportion of registered population aged 85+ years	0.022	0.012	0.014	0.021	0.029
Income deprivation score	0.176	0.110	0.087	0.150	0.245
Proportion of registered patients of UK white ethnicity	0.764	0.254	0.649	0.879	0.949
Proportion of registered patients unemployed	0.051	0.047	0.017	0.039	0.072
Response rate to General Practitioner Patient Survey	0.412	0.110	0.330	0.419	0.496
Atrial fibrillation prevalence (proportion of registered patients)	0.020	0.254	0.011	0.017	0.021
COPD prevalence (proportion of registered patients)	0.027	0.669	0.013	0.018	0.024
Asthma prevalence (proportion of registered patients)	0.078	1.660	0.051	0.059	0.067
Heart failure prevalence (proportion of registered patients)	0.009	0.138	0.005	0.007	0.009
Coronary heart disease prevalence (proportion of registered patients)	0.042	0.853	0.025	0.032	0.040
Distance from GP practice to nearest type one ED (km)	6.858	6.677	2.503	4.497	9.014
Rate of attendance at type 1–3 EDs (per 10 patients)	0.830	1.012	0.105	0.388	1.253
Number of GPs	5.349	3.388	3.000	4.915	7.099
Variables included in sensitivity analyses					
Rated good or outstanding by CQC on first inspection	0.828	0.378	1.000	1.000	1.000
Weighted proportion of patients who could see their preferred GP	0.590	0.171	0.469	0.595	0.715

COPD, chronic obstructive pulmonary disease; CQC, Care Quality Commission; ED, emergency department; GP, general practitioner.

Average practice achievement of clinical QOF points was 96% (table 1). On average, 78% of patients would recommend their practice; 85% reported good overall experience of their GP surgery; 62% were able to see their preferred GP; 73% found it easy to get through to their practice on the phone; 85% were able to make an appointment; and 37% of patients were able to get a same-day appointment.

The associations between attendance volumes and practice and population characteristics are presented in table 2. A higher proportion of registered patients aged 0–4 years, higher levels of deprivation and higher unemployment are all associated with higher rates of attendances across all four measures. Distance to the nearest ED, the response rate to the GPPS and the rate of attendance at other emergency care facilities are negatively associated with ED attendances across all four measures. Higher proportions of registered patients aged 75–84 and 85 and over are associated with higher rates of total attendances but not with any of the three measures of avoidable attendances.

**Table 2:** Associations between ED attendance volumes and practice and population characteristics

	All attendances	Self-referred discharged attendances	GP treatable attendances	No treatment and no follow-up attendances
	IRR (95% CI)	IRR (95% CI)	IRR (95% CI)	IRR (95% CI)
Proportion of patients aged 0–4†	1.444*** (1.316 to 1.584)	1.501*** (1.258 to 1.791)	1.390** (1.118 to 1.728)	1.444** (1.096 to 1.904)
Proportion of patients aged 5–15†	0.966 (0.910 to 1.025)	1.086 (0.982 to 1.202)	1.091 (0.956 to 1.247)	1.242* (1.050 to 1.469)
Proportion of patients aged 45–54†	1.159** (1.061 to 1.266)	1.089 (0.923 to 1.285)	1.074 (0.895 to 1.290)	0.973 (0.782 to 1.211)
Proportion of patients aged 55–64†	0.990 (0.903 to 1.085)	1.050 (0.893 to 1.236)	0.966 (0.809 to 1.154)	1.023 (0.816 to 1.283)
Proportion of patients aged 65–74†	0.979 (0.885 to 1.082)	0.990 (0.804 to 1.218)	0.824 (0.627 to 1.082)	0.831 (0.577 to 1.195)
Proportion of patients aged 75–84†	1.194** (1.059 to 1.346)	1.126 (0.889 to 1.426)	1.349 (0.971 to 1.872)	1.260 (0.862 to 1.844)
Proportion of patients aged 85 and over†	1.396*** (1.207 to 1.615)	0.946 (0.768 to 1.165)	0.980 (0.651 to 1.474)	0.984 (0.663 to 1.462)
Income deprivation score†	1.044*** (1.030 to 1.057)	1.029* (1.006 to 1.052)	1.052** (1.021 to 1.084)	1.045** (1.013 to 1.078)
Rural practice	1.055*** (1.023 to 1.089)	1.108*** (1.043 to 1.176)	1.065 (0.996 to 1.139)	1.070 (0.987 to 1.160)
Number of GPs	0.999 (0.997 to 0.002)	0.995 (0.990 to 1.000)	0.998 (0.992 to 1.005)	0.993 (0.986 to 0.000)
Proportion of patients of UK white ethnicity†	1.016* (1.001 to 1.031)	1.020 (0.990 to 1.052)	1.025 (0.990 to 1.062)	1.062* (1.014 to 1.113)
Proportion of patients unemployed†	1.101*** (1.060 to 1.144)	1.072*** (1.031 to 1.115)	1.137*** (1.072 to 1.205)	1.119*** (1.060 to 1.181)
Atrial fibrillation prevalence§	0.957* (0.925 to 0.990)	0.962 (0.898 to.030)	0.939 (0.873 to 1.011)	0.945 (0.864 to 1.034)
COPD prevalence§	1.046*** (1.028 to 1.063)	1.048** (1.013 to 1.084)	1.041 (0.999 to 1.085)	1.047 (0.996 to 1.100)
Asthma prevalence§	0.988** (0.979 to 0.996)	0.985* (0.971 to 0.999)	1.002 (0.982 to 1.023)	0.985 (0.963 to 1.007)
Heart failure prevalence§	1.005 (0.960 to 1.053)	0.971 (0.900 to 1.048)	0.987 (0.904 to 1.078)	0.929 (0.835 to 1.034)
Coronary heart disease prevalence§	1.002 (0.985 to 1.018)	1.009 (0.975 to 1.045)	0.984 (0.940 to 1.031)	1.023 (0.969 to 1.080)
Rate of attendance at type 2–4 EDs	0.928*** (0.906 to 0.950)	0.834*** (0.793 to 0.876)	0.824*** (0.775 to 0.876)	0.775*** (0.720 to 0.834)
Distance to nearest ED	0.983*** (0.979 to 0.986)	0.970*** 0.962 to 0.977)	0.974*** (0.966 to 0.982)	0.975*** (0.965 to 0.985)
Responses to GPPS†	0.931*** (0.911 to 0.952)	0.933** (0.890 to 0.977)	0.921** (0.867 to 0.978)	0.913** (0.853 to 0.977)
N	7521	7521	7521	7521

*P< 0.05, **P<0.01, ***P<0.001.

†These variables are scaled so that the IRRs reflect the effects of 10 percentage point differences.

§These variables are scaled so that the IRRs reflect the effects of 1 percentage point difference. Models also include measures of primary care quality, coefficients for which are presented in table 3. They also include indicators for the 13 NHS England local offices in which a general practice is located (NHS England region: London (n=1354), Wessex (n=303), Cheshire and Merseyside (n=379), Cumbria and North East (n=450), Lancashire and Greater Manchester (n=704), Yorkshire and Humber (n=743), Central Midlands (n=550), East (n=533), North Midlands (n=489), West Midlands (n=653), South Central (n=414), South East (n=563) and South West (n=386)).

COPD, chronic obstructive pulmonary disease; ED, emergency department; GP, general practitioner; GPPS, General Practitioner Patient Survey; IRR, incidence rate ratio.

The associations between attendance volumes and the primary care quality measures from the same models are presented in table 3. The proportion of patients reporting easy phone access and the proportion of patients able to make an appointment both demonstrate a significant negative association with rates of attendances across all four attendance measures. The magnitude of the relationship between attendance rates and these two measures of access increases as the strictness of the definition of avoidable attendances increases, from total attendance volumes down to ‘no treatment and no follow-up’ avoidable attendances. This indicates that measures of avoidable attendances are more sensitive to primary care access than total attendance volumes. Total attendances and ‘self-referred discharged’ avoidable attendances were also found to be negatively associated with primary care quality as measured by clinical QOF scores. The magnitude of the association is stronger for ‘self-referred discharged’ avoidable attendances than for total ED attendances.

**Table 3:** Associations between ED attendance volumes and primary care quality

	All ED attendances	Self-referred discharged attendances	GP treatable attendances	No treatment and no follow-up attendances
	IRR (95% CI)	IRR (95% CI)	IRR (95% CI)	IRR 95% CI)
Clinical QOF score achievement†	0.987* (0.975 to 0.998)	0.977* (0.954 to 1.000)	0.982 (0.950 to 1.014)	0.966 (0.924 to 1.010)
Proportion of patients that would recommend the practice†	0.995 (0.983 to 1.007)	0.978 (0.954 to 1.002)	0.978 (0.951 to 1.006)	0.978 (0.943 to 1.015)
Proportion of patients reporting good overall experience†	1.007 (0.989 to 1.026)	1.014 (0.983 to 1.047)	1.020 (0.984 to 1.058)	1.018 (0.973 to 1.065)
Proportion of patients that could see their preferred GP†	0.998 (0.993 to 1.004)	1.006 (0.994 to 1.017)	1.005 (0.993 to 1.018)	1.012 (0.996 to 1.029)
Proprtion of patients reporting easy phone access†	0.987*** (0.981 to 0.994)	0.985* (0.970 to 0.999)	0.978** (0.963 to 0.994)	0.976* (0.955 to 0.998)
Proprtion of patients that were able to get an appointment to see or speak to someone†	0.978*** (0.967 to 0.990)	0.963** (0.941 to 0.985)	0.963* (0.934 to 0.993)	0.934*** (0.898 to 0.971)
Proportion of patients that were able to get a same day appointment†	0.997 (0.991 to 1.003)	0.992 (0.981 to 1.004)	0.992 (0.977 to 1.006)	0.990 (0.972 to 1.009)
N	7521	7521	7521	7521
Pseudo-R^2^	0.045	0.039	0.047	0.042
Number of primary care quality sensitive attendances	346 334	187 815	74 464	44 002
Proportion of attendances sensitive to primary care quality (%)	2.48	3.70	4.10	5.26

*P< 0.05, **P< 0.01, ***P< 0.001. The reported pseudo-R^2^ statistic is McFadden’s pseudo R^2^ calculated as one minus the ratio of the log-likelihood of the estimated model to the log-likelihood of a model containing no covariates.

†These variables are scaled so that the IRRs reflect the effects of 10 percentage point differences. Models also include practice and population characteristics, coefficients for which are presented in table 2 and indicators for the 13 NHS England local offices in which a general practice is located (NHS England region: London (n=1354), Wessex (n=303), Cheshire and Merseyside (n=379), Cumbria and North East (n=450), Lancashire and Greater Manchester (n=704), Yorkshire and Humber (n=743), Central Midlands (n=550), East (n=533), North Midlands (n=489), West Midlands (n=653), South Central (n=414), South East (n=563); South West (n=386)).

ED, emergency department; GP, general practitioner; IRR, incidence rate ratio; QOF, Quality and Outcomes Framework.

We estimate that levels of primary care quality below the national average are associated with 2.48% of total ED attendances, 3.70% of self-referred discharged attendances, 4.10% of GP treatable attendances and 5.26% no treatment and no follow-up attendances. However, when we estimate the absolute number of attendances that could potentially be avoided if levels of quality that were below the average were raised to the average, our models generate values of 346 334 total attendances, 187 815 self-referred discharged attendances, 74 464 GP treatable attendances and 44 002 no treatment and no follow-up attendances. While the relative association between primary care quality and attendances strengthens as stricter definitions of avoidability are applied, our estimates of the absolute number of attendances that could potentially be avoided suggests some attendances outside of these definitions of avoidable are nevertheless sensitive to primary care quality.

The results were robust to three sensitivity analyses (online supplemental appendix 4). Removing practices within 20 km of the borders with Scotland or Wales did not notably change the magnitude or implications of the results. CQC inspection ratings were not found to be statistically significantly associated with the volume of attendances. Using the weighted proportion of patients able to see their GP did not change the results.

## Discussion

### Summary of findings

Improvements in primary care quality are often proposed as a solution to the problem of continually increasing ED attendances. However, the relationship between quality of primary care and the demand for ED services is poorly understood. We find that regardless of whether we examine all attendances or narrow down to definitions of an avoidable attendance, lower attendance volumes are associated with better access to primary care services in the form of ability to get an appointment and ease of telephone contact. This relationship exists even after controlling for a wide range of practice and population characteristics. However, we do not find attendance volumes to be associated with patient experience or the proportion of patients able to see their preferred GP, which is often interpreted as a measure of care continuity.

There is a lack of agreement in the literature as to what constitutes an avoidable ED attendance, and this has resulted in estimates of the proportion of ED use classified as inappropriate ranging from 10% to 90%.^8^ We compared across three commonly used definitions of an avoidable attendance to examine whether some were more sensitive than others to primary care quality. We found that the magnitudes of the associations with indicators of primary care quality were larger for stricter definitions of avoidable attendances. Stricter measures of avoidable attendances are therefore more sensitive to primary care quality in relative terms.

However, the magnitudes of these associations were small. We found that less than 3% of total ED attendances were associated with levels of primary care quality that were below the national average. Even when applying the strictest definition of an avoidable attendance (no treatment and no follow-up), we found that only 5.3% of these attendances were associated with levels of primary care quality below the national average. These results suggest that ED attendances may be far less responsive to changes in primary care quality than is implied by previous estimates of the prevalence of avoidable attendances. This may in turn explain the failure of many initiatives to reduce ED attendances through improvements to primary care.

While the proportion of attendances that are sensitive to primary care quality increases with the strictness of the definition, the absolute number of primary care sensitive attendances falls, from 346 000 of all attendances to 188 000 self-referred discharged attendances, 75 000 GP treatable attendances, and 44 000 attendances with no treatment and no follow-up. This suggests that while the attendances characterised by the stricter definitions are more likely to be avoidable, some attendances not meeting these avoidability criteria are nevertheless sensitive to primary care quality. Focusing only on attendances meeting these avoidability criteria will therefore miss some attendances that are sensitive to primary care quality.

A potential explanation for this finding lies in the definitions of avoidable attendances commonly used in the literature and applied in this paper. A recent classification suggests that there are three distinct categories of avoidable ED attendances, classified based on the care that was required: clinically divertable, clinically preventable and clinically unnecessary attendances.^14^ The definitions of an avoidable attendance that we have applied here encompass a combination of clinically divertable and clinically unnecessary attendances. However, none of the definitions capture clinically preventable attendances.

The omission of clinically preventable attendances from the definitions of avoidable attendances may therefore explain why we find that some attendances outside of the applied definitions of avoidable are nevertheless sensitive to primary care quality. While the phenomenon of preventability through earlier intervention in primary care has received wide attention when patients require admission to the hospital in the form of ambulatory care-sensitive conditions,^28^ this notion of preventability is largely absent in definitions of avoidable ED attendances.

In the absence of definitions able to capture all three categories of avoidable ED attendances empirically, it may be preferable to examine total attendance volumes. When evaluating the impact of interventions aimed at improving primary care quality, while a stricter definition of avoidable attendances may provide a stronger signal, examining total ED attendances will capture a greater absolute number of responsive attendances.

### Strengths and limitations

We used national data covering all ED attendances in England over the period of a year. We applied three different definitions of avoidable attendances used in previous literature, in addition to examining total attendance volumes, and examined the sensitivity of these volumes to seven different measures of primary care quality covering access, patient experience and clinical quality.

While we attempted to include a wide variety of quality measures, information on waiting times and rates of use were not available. We therefore had to rely on patient-reported measures of access to primary care. Our ability to detect significant associations between attendances and primary care quality was constrained by the range of variations in quality in these variables. It is also important to recognise that the variables we use to measure quality contain measurement error, particularly those from the GPPS. This too will have affected our ability to measure the associations between attendances and primary care quality. It will also mean that some of the below-average quality that we include in our impact measurement will have occurred only by chance.

We controlled for a wide range of practice and population characteristics that may have confounded the relationships between quality and the volume of avoidable attendances, including the prevalence of five chronic conditions, but were unable to control for rates of multimorbidity because these are not available from national data.^29^

Our calculations were made on the basis of cross-sectional associations, which are likely to overestimate the true causal magnitude of the relationships. Furthermore, raising the quality of primary care services to current average levels at a minimum across England would represent a huge achievement, far larger than any previous quality improvement programme. Although some aspects of primary care quality have been declining over recent years,^30^ these declines are substantially smaller than the changes in quality that we consider when raising below-average quality to national average levels. For example, the percentage of GPPS responders who report very or fairly easy telephone access in 2015/2016 was five percentage points lower than what has been previously reported for the period 2011/2012– 2013/2014.^31^ By way of comparison, the average increase in quality that we considered when raising below-average quality to national average levels on this same access measure was 15.8 percentage points. Furthermore, while the use of the contemporaneous national average is a somewhat arbitrary benchmark, we do not consider a national quality deficit to be a plausible explanation for our results.

### Relation to existing literature

Our finding that practice-level ED attendance volumes are sensitive to access to primary care services is consistent with existing research.^4^ However, previous studies have generally only examined the relationship using one measure of ED attendances^5–7 32^ and/or a single dimension of primary care quality.^7 32^ Our more comprehensive examination confirms previous findings that attendance volumes are not associated with overall patient experience,^7^ but reveals that ED attendances are sensitive to measures of primary care access and clinical quality. There remains a discrepancy between the findings on the relationship between access and use at the patient level^31 33^ and at the practice level,^5–7 32^ which should be a priority for future research.

The concept of avoidable ED attendances has been operationalised through consultation with clinical experts who have identified characteristics of attendances which could indicate that patients did not require ED care.^34 35^ Our results suggest that while these criteria identify the types of attendances that are more sensitive to primary care quality, over 90% of attendances identified as potentially avoidable are not associated with below-average primary care quality regardless of which definition of avoidable is used. Some attendances classified as avoidable because the patient did not require any clinical care are in fact driven by complex social problems.^14^ Further research is needed to explore the sensitivity of ED attendance volumes to social and welfare services, and to investigate potential solutions outside of the healthcare system.

### Policy implications

Although the volume of attendances typically thought to indicate an avoidable attendance is sensitive to primary care quality, the proportion of attendances associated with poor quality of primary care services is much smaller than the numbers suggested by previous prevalence studies and in policy documents.

The potential reduction in ED attendance volumes that could therefore be achieved through quality improvement in primary care is likely to be small.

## Data Availability

Data may be obtained from a third party and are not publicly available. Data are either publicly available online from NHS Digital or are available upon request from NHS Digital.
