# Supplementary material for: How sensitive are avoidable emergency department attendances to primary care quality? Retrospective observational study
Source: BMJ Qual Saf. 2020 Nov 3;30(11):884–92. doi: 10.1136/bmjqs-2020-011651 (PMC8543208; doi:10.1136/bmjqs-2020-011651)
Supplement: Supplementary data [file bmjqs-2020-011651supp001.pdf]

Appendix 1 Descriptions of published definitions of avoidable attendances and the Hospital Episode Statistics Accident &Emergency data variable codes used to identify them

Definition	Source	Description	Hospital Episode Statistics Accident &Emergency variable codes
1: Self-referred discharges	Tammes et al (1)	Self-referred discharged Emergency Department attendance.	AEREFSOURCE 01 = Self-referral;<br>AEATTENDDISP 03 = Discharged – did not require any follow-up treatment, 12 = Left department before being treated, 13 = Left department having refused treatment, or 02 = Discharged – follow-up treatment to be provided by general practitioner.
2: GP treatable	NHS Digital (2)	First attendance with some recorded treatments or investigations all of which may have been reasonably provided by a GP, followed by discharge home or to GP care.	INVEST_N: 06 = Urinalysis=, 21 = Pregnancy test, 22 = Dental investigation, or 24 = None;<br>TREAT_N: 221 = Guidance/advice only - written, 222 = Guidance/advice only - verbal, 30 = Recording vital signs, 56 = Dental treatment, 57 = Prescription/medicines prepared to take away, 99 = None, or 07 = Prescriptions;<br>AEATTENDDISP: 02 = Discharged - follow-up treatment to be provided by general practitioner, 03 = Discharged - did not require any follow-up treatment, or 12 = Left department before being treated;<br>AEARRIVALMODE: all codes except for 1 = non-ambulance arrivals.
3: No treatment and no follow-up	McHale et al (3)	Patients who were self-referred; were not attending a follow-up; received no investigation and either no treatment or ‘guidance/advice only’; and were discharged with either no follow-up or follow-up with primary care.	AEREFSOURCE: 01 = Self-referral;<br>AEATTENDCAT: 1 = First Accident and Emergency attendance, or 3 = Follow-up Accident and Emergency attendance - unplanned;<br>INVEST_N: 24 = None;<br>TREAT_N: 221 = Guidance/advice only - written, 222 = Guidance/advice only - verbal, or 99 = None,<br>AEATTENDDISP: 02 = Discharged - follow-up treatment to be provided by general practitioner, or 03 = Discharged - did not require any follow-up treatment

Parkinson B, *et al. BMJ Qual Saf* 2021; 30:884–892. doi: 10.1136/bmjqs-2020-011651

Appendix 2: Data management

We applied the following process to generate annual practice-level counts of each type of avoidable emergency department attendance from the patient-level attendances data set:

Of the 14,864,083 attendances at type 1 emergency departments in England during the financial year 1st April 2015 to 31st March 2016, 64,706 (0.4%) were recorded as having no registered GP. 480,705 (3.2%) attendances were recorded as having a general practice code that was not applicable, not known, or had no practice code submitted. A further 137,274 (0.9%) of attendances had a practice code that did not belong to the master list of 7,758 practices, obtained from practice registrations data from July 2015 (4). This left n=14,181,398 attendances which we were able to link to the general practice with which the patient is registered. These attendances were then aggregated to practice-level counts of total emergency department attendances and counts of the three definitions of avoidable attendances.

Data on the practice-level counts of emergency department attendances were then merged with practice factors using GP practice identifier codes as follows: The master file of practice codes, the number of registered patients, and age-gender distribution of patients were obtained from NHS Digital (n=7,758 general practices) (4). Practices with a registered population of less than 1,000 patients were dropped (n=49). We also drop practices that were established after 1st April 2015 (n=1) and those that closed before 1st April 2016 (n=5), using practice open and close dates obtained from NHS Digital (5), since these practices were not active for the full financial year we examine. Furthermore, we drop practices that serve atypical populations (n=16), such as those that solely register students, homeless people and asylum seekers. This was done by searching practice names for the terms 'Student', 'University', 'Homeless', 'Asylum' and 'Vulnerable'. These restrictions leave n=7,687 general practices. Using this master list of general practices we then merge in attendance counts (not merged n=0), GP Patient Survey (GPPS) data (not merged n=2), and Quality and Outcomes Framework (QOF) data (not merged n=127). We exclude practices that are missing any of the covariates in the analysis (n=37), leaving a final sample of 7,521 general practices.

Appendix 3: Description of the explanatory variables included in our analysis and the data from which these were sourced

Variable	Description	Source
**Primary care quality measures**		
Clinical quality	Proportion of total clinical Quality and Outcomes Framework (QOF) score achieved	Quality and Outcomes Framework (6)
Recommend the practice	Proportion of patients answering 'Yes, would definitely recommend' or 'Yes, would probably recommend' to the question 'Would you recommend your GP surgery to someone who has just moved to your local area?' vs 'Not sure', 'No, would probably not recommend', 'No, would definitely not recommend', or 'Don't know'.	GP Patient Survey (7)
Good overall experience	Proportion of patients answering 'Very good or 'Fairly good' to the question 'Overall, how would you describe your experience of your GP surgery?' vs 'neither good nor poor', 'fairly poor', or 'very poor'.	GP Patient Survey (7)
See their preferred GP	Proportion of patients answering 'Always or almost always' or 'A lot of the time' to the question 'How often do you see or speak to the GP you prefer? vs 'some of the time', 'never or almost never' or 'not tried at this GP surgery' [among patients with preferred GP].	GP Patient Survey (7)
Easy phone access	Proportion of patients answering 'Very easy' or 'Fairly easy' to the question 'Generally, how easy is it to get through to someone at your GP surgery on the phone?' vs 'Not very easy', 'Not at all easy', or 'Haven't tried'	GP Patient Survey (7)
Able to get an appointment to see or speak to someone	Proportion of patients answering 'Yes' or 'Yes, but had to call back closer to the day I wanted the appointment' to the question 'Were you able to get an appointment to see or speak to someone?' vs 'No' and 'Can't remember' [at the last time they wanted to see or speak to a GP or nurse from their GP surgery]	GP Patient Survey (7)
Able to get a same day appointment	Proportion of patients answering 'On the same day' to the question 'How long after initially contacting the surgery did you actually see or speak to them?' vs 'on the next working day', 'a few days later', 'a week or more later', or 'can't remember' [at the last time they wanted to see or speak to a GP or nurse from their GP surgery].	GP Patient Survey (7)
**Practice characteristics**		
List size	Total number of patients registered at a practice as at July 2015	NHS Digital, Number of patients registered at a GP Practice (4)
Proportion of list size aged 0-4 years	Proportion of total list size aged 0-4 years at a practice as at July 2015	NHS Digital, Number of patients registered at a GP Practice (4)
Proportion of list size aged 5-15 years	Proportion of total list size aged 5-15 years at a practice as at July 2015	NHS Digital, Number of patients registered at a GP Practice (4)
Proportion of list size aged 16-44 years	Proportion of total list size aged 16-44 years at a practice as at July 2015	NHS Digital, Number of patients registered at a GP Practice (4)
Proportion of list size aged 45-54 years	Proportion of total list size aged 45-54 years at a practice as at July 2015	NHS Digital, Number of patients registered at a GP Practice (4)
Proportion of list size aged 55-64 years	Proportion of total list size aged 55-64 years at a practice as at July 2015	NHS Digital, Number of patients registered at a GP Practice (4)
Proportion of list size aged 65-74 years	Proportion of total list size aged 65-74 years at a practice as at July 2015	NHS Digital, Number of patients registered at a GP Practice (4)
Proportion of list size aged 75-84 years	Proportion of total list size aged 75-84 years at a practice as at July 2015	NHS Digital, Number of patients

		registered at a GP Practice (4)
Proportion of list size aged 85+ years	Proportion of total list size aged 85+ years at a practice as at July 2015	NHS Digital, Number of patients registered at a GP Practice (4)
Income deprivation	2015 Index of Multiple Deprivation (IMD) income domain score of the Lower Super Output Area (LSOA) within which the general practice is located	Ministry of Housing, Communities & Local Government. English indices of deprivation 2015 (8)
Rurality	Classifies GP practices as either 'Rural' or 'Urban' based on the LSOA of the practice	Office for National Statistics, Rural Urban Classification (2011) of Lower Layer Super Output Areas in England and Wales (9)
NHS Region	Classifies GP practices into the 13 NHS England Regions based on their Clinical Commissioning Group	ONS Clinical Commissioning Group to NHS Region (Geography) to NHS Commissioning Region (July 2015) Lookup in England (10)
Ethnicity	Proportion of respondents who answered 'UK White' to 'What is your ethnic group?'	GP patient survey (7)
Unemployment	Proportion of respondents who answered 'Unemployed' to 'Which of these bests describes what you are doing at present?'	GP patient survey (7)
GP Patient Survey (GPPS) response rate	Percentage of returned surveys as a proportion of total surveys sent	GP patient survey (7)
Atrial Fibrillation prevalence	Number of patients with an 'initial event'; paroxysmal (intermittent); persistent and permanent atrial fibrillation, as a proportion of total patients registered at the practice	Quality and Outcomes Framework (11)
Chronic Obstructive Pulmonary Disease prevalence	Number of patients with a diagnosis of chronic obstructive pulmonary disease, as a proportion of total patients registered at the practice	Quality and Outcomes Framework (11)
Asthma prevalence	Number of patients with a diagnosis of asthma, as a proportion of total registered patients	Quality and Outcomes Framework (11)
Heart Failure prevalence	Number of patients with diagnosis of heart failure, as proportion of total registered patients	Quality and Outcomes Framework (11)
Coronary Heart Disease prevalence	Number of patients with a diagnosis of coronary heart disease, as a proportion of total registered patients	Quality and Outcomes Framework (11)
Distance from practice to closest emergency department	Geodetic distance in kilometres between practice postcode and closest type 1 emergency department.<br>Postcodes and corresponding coordinates of GP practices and emergency departments were obtained and Stata's 'geonear' command was used to calculate distance to the nearest emergency department from each GP practice and the corresponding geodetic distance. When an emergency department opened or closed in 2015/16, we used the distance between each GP practice and emergency department at the start and end of the year, weighted by the proportion of the year each emergency department was open.	Practice postcodes: NHS digital, GP Practices (epraccur) (5)<br>Emergency department postcodes: department of health (12) and NHS digital (13).<br>ONS Postcode directory (14).
Use/availability of alternative urgent care services	Rate of attendance at type 1-3 emergency departments per 1,000 registered patients	Hospital episode statistics
Total number of GPs	The average number of GPs working at the practice within 2015/16.<br>Data contains a list of all GPs that have worked in general practice, and the periods that they have worked at each practice. We calculated the number of days that each GP worked at each practice in each year. We then summed these periods for each practice-	NHS Digital, GPs by GP practice (epracmem) (15)

year to calculate the average number of GPs working at the practice across the days of that year. We were unable to use NHS Digitals' full-time equivalent GP data for the 2015/16 financial year due to the amount of missing data and changes in the methodolgy for calculating FTEs in 2015/16.

Parkinson B, *et al. BMJ Qual Saf* 2021; 30:884–892. doi: 10.1136/bmjqs-2020-011651

Appendix 4: Sensitivity analysis: associations between total emergency department attendances and primary care quality

	I<br>Removing practices within 20km Scottish & Welsh boarder	II<br>Including CQC inspection rating as additional practice variable	III<br>Including unweighted 'see preferred GP' variable for practices with complete data for weighted variable	III<br>Including weighted 'see preferred GP' variable for practices with complete data
Proportion of clinical QOF score achievement	0.988* [0.00597]	0.982** [0.00635]	0.988* [0.00583]	0.988* [0.00582]
Proportion that would recommend the practice	0.995 [0.00638]	0.992 [0.00637]	0.988 [0.00626]	0.989 [0.00625]
Proportion reporting good overall experience	1.008 [0.00966]	1.008 [0.00907]	1.013 [0.00925]	1.013 [0.00928]
Proportion that could see their preferred GP	0.998 [0.00296]	0.999 [0.00288]	0.998 [0.00285]	0.997 [0.00271]
Proportion reporting easy phone access	0.987*** [0.00338]	0.988*** [0.00311]	0.988*** [0.00331]	0.988*** [0.00330]
Proportion able to get an appointment to see or speak to someone	0.977*** [0.00604]	0.982** [0.00560]	0.980** [0.00604]	0.981** [0.00607]
Proportion able to get a same day appointment	0.997 [0.00293]	0.996 [0.00289]	0.995 [0.00287]	0.995 [0.00288]
Practice received CQC rating good or outstanding	- -	0.991 [0.00922]	- -	- -
N	7189	7209	7124	7124
pseudo R^2	0.045	0.047	0.046	0.046
Primary care quality sensitive attendances	338,798	335,058	341,947	350.183
Proportion of attendances which are sensitive to PC	2.52%	2.46%	2.47%	2.53%

Notes: Standard errors clustered at Clinical Commissioning Group level in brackets. Models also include practice and population characteristics and indicators for the 13 NHS England local offices in which a general practice is located (NHS England Region: London (N=1,354); Wessex (N=303); Cheshire and Merseyside (N=379); Cumbria and North East (N=450); Lancashire and Greater Manchester (N=704); Yorkshire and Humber (N=743); Central Midlands (N=550); East (n=533); North Midlands (N=489); West Midlands (N=653); South Central (N= 414); South East (N=563); South West (N=386)). * $p < 0.05$, ** $p < 0.01$, *** $p < 0.001$.

Sources

1. Tammes P, Morris RW, Brangan E, Checkland K, England H, Huntley A, et al. Exploring the relationship between general practice characteristics, and attendance at walk-in centres, minor injuries units and EDs in England 2012/2013: a cross-sectional study. Emerg Med J EMJ. 2016 Oct;33(10):702–8.

2. NHS Digital. Unnecessary A and E attendances [Internet]. NHS Digital. [cited 2018 May 25]. Available from: https://digital.nhs.uk/data-and-information/data-tools-and-services/data-services/innovative-uses-of-data/demand-on-healthcare/unnecessary-a-and-e-attendances

3. McHale P, Wood S, Hughes K, Bellis MA, Demnitz U, Wyke S. Who uses emergency departments inappropriately and when - a national cross-sectional study using a monitoring data system. BMC Med. 2013 Dec 13;11:258.

4. NHS Digital. Numbers of Patients Registered at a GP Practice - July 2015 [Internet]. 2015 [cited 2019 Apr 17]. Available from: https://digital.nhs.uk/data-and-information/publications/statistical/patients-registered-at-a-gp-practice/july-2015

5. NHS Digital. GP and GP practice related data: GP Practices (epraccur) [Internet]. NHS Digital; 2018 May [cited 2018 Jul 20]. Available from: https:/https://digital.nhs.uk/services/organisation-data-service/data-downloads/gp-and-gp-practice-related-data/www.england.nhs.uk/statistics/statistical-work-areas/gp-patient-survey/

6. NHS Digital. QOF 2015-16: Achievements at practice level, all domains [Internet]. 2016. Available from: https://files.digital.nhs.uk/publicationimport/pub22xxx/pub22266/qof-1516-prac-dom-ach.xlsx

7. Ipsos MORI. GP Patient Survey. July 2016. Practice data (weighted). [Internet]. 2016. Available from: https://www.gp-patient.co.uk/surveysandreports-10-16

8. Ministry of Housing, Communities & Local Government. English indices of deprivation 2015 [Internet]. GOV.UK. 2015 [cited 2018 Jul 30]. Available from: https://www.gov.uk/government/statistics/english-indices-of-deprivation-2015

9. Office for National Statistics O for N. Rural Urban Classification (2011) of Lower Layer Super Output Areas in England and Wales [Internet]. 2018 [cited 2019 Oct 17]. Available from: https://data.gov.uk/dataset/b1165cea-2655-4cf7-bf22-dfbd3cdeb242/rural-urban-classification-2011-of-lower-layer-super-output-areas-in-england-and-wales

10. Clinical Commissioning Group to NHS Region (Geography) to NHS Commissioning Region (July 2015) Lookup in England [Internet]. 2015 [cited 2019 Oct 17]. Available from: https://geoportal.statistics.gov.uk/items?tags=Local%20Commissioning%20Group

11. NHS Digital. QOF 2015-16: Prevalence, all groups at all levels v2 [Internet]. NHS Digital; 2016 Oct [cited 2018 Jul 20]. Available from: https://files.digital.nhs.uk/publicationimport/pub22xxx/pub22266/qof-1516-prev-all-lev-v2.xlsx

12. Department of Health. Address of Major A&E departments in England and Wales. (Obtained under the Freedom of Information Act (2000) from The Department of Health; 26th October 2012; reference DE00000729700) [Internet]. 2012 [cited 2019 Nov 3]. Available from: https://www.whatdotheyknow.com/request/address_of_major_ae_departments

13. NHS Digital. Addresses of major (type 1) A&E departments in England. (Obtained under the Freedom of Information Act (2000) from NHS Digital; 12 April 2017; Reference NIC-98262-D1L6S) [Internet]. 2017 [cited 2019 Nov 3]. Available from: https://www.whatdotheyknow.com/request/address_of_major_ae_departments

14. Office for National Statistics. ONS Postcode Directory (May 2016) [Internet]. 2017 [cited 2019 Feb 1]. Available from: https://data.gov.uk/dataset/bf86a637-7e4e-43df-ac96-2cb4670f799b/ons-postcode-directory-may-2016

15. NHS Digital. GP and GP practice related data: GPs by GP Practices (epracmem) [Internet]. 2018 May [cited 2018 Jul 20]. Available from: https://digital.nhs.uk/services/organisation-data-service/data-downloads/gp-and-gp-practice-related-data

Parkinson B, *et al. BMJ Qual Saf* 2021; 30:884–892. doi: 10.1136/bmjqs-2020-011651
